# A Retrospective Study of Predictive Factors and Functional Outcomes of Medial Opening Wedge High Tibial Osteotomy for Varus Medial Compartment Knee Osteoarthritis: Insights From a Resource-Limited Setting

**DOI:** 10.7759/cureus.79377

**Published:** 2025-02-20

**Authors:** Abdulrakib Almirah, Anwar Mahyoub, Khaled Swailem, Wael Alhamadi, Abdulfattah Altam

**Affiliations:** 1 Department of Orthopedics, Faculty of Medicine, Sana'a University of Medical Sciences, Sana'a, YEM; 2 Department of Orthopedics, School of Medicine, 21 September University for Medical and Applied Sciences, Sana'a, YEM; 3 Department of General Surgery, 21 September University for Medical and Applied Sciences, Sana'a, YEM

**Keywords:** functional outcome, knee osteoarthritis, medial opening wedge high tibial osteotomy, predictive factors, varus medial compartmental

## Abstract

Introduction: Medial opening wedge high tibial osteotomy (MOWHTO) represents a well-established surgical intervention for varus malalignment associated with medial compartment knee osteoarthritis (KOA). Given the increasing prevalence of KOA, particularly within aging populations, a thorough evaluation of effective therapeutic strategies is paramount. This study aimed to identify predictors of MOWHTO failure and assess the functional outcomes of this procedure in a resource-limited setting, thereby informing clinical decision-making and optimizing patient care.

Materials and methods: This retrospective study analyzed the outcomes of MOWHTO performed on adult patients diagnosed with KOA and varus deformity at two hospitals in Sana'a, Yemen, between October 2019 and April 2023. Data pertaining to demographics, preoperative characteristics, intraoperative details, radiographic findings, and postoperative parameters were meticulously collected and analyzed. Clinical outcomes were evaluated using the American Knee Society Functional Score (AKSFS), the Clinical American Knee Society Score (CAKSS), and the Western Ontario and McMaster Universities (WOMAC) Osteoarthritis Index. Multivariate regression analysis determined the associations between patient-specific factors and MOWHTO failure, defined as conversion to unicompartmental or total knee arthroplasty.

Results: A cohort of 102 patients underwent MOWHTO, with a mean follow-up duration of 24.4 ± 7.0 months. The mean age of the patient population was 42.6 ± 8.2 years, with a female predominance (n=69, 67.6%). Disease severity, as per the Kellgren-Lawrence classification, was graded as follows: grade 1 in 19 (18.6%) knees, grade 2 in 48 (47.1%) knees, grade 3 in 22 (21.6%) knees, and grade 4 in 13 (12.7%) knees. Postoperatively, statistically significant improvements were observed in the mean AKSFS, CAKSS, and WOMAC scores at the final follow-up compared to preoperative values (all p<0.05). Post-surgical complications included pin-tract infections (n=5, 4.9%) and lateral cortex fractures (n=4, 3.9%). Overall, 92 (90.2%) knees demonstrated successful outcomes, while 10 (9.8%) were classified as failures. Multivariate analysis revealed that advanced age (OR: 1.89; 95% CI: 1.02-3.50, p=0.043) and higher body mass index (BMI) (OR: 2.40; 95% CI: 1.13-5.10, p = 0.022) were significant predictors of surgical failure.

Conclusions: This study confirms that MOWHTO improves clinical outcomes for varus medial compartment KOA, especially in younger patients with a normal BMI, demonstrating its potential in resource-limited settings. These findings highlight the importance of considering age and BMI in treatment decisions; future prospective research is warranted to refine patient selection criteria and expand the applicability of MOWHTO.

## Introduction

Knee osteoarthritis (KOA) affects around 250 million individuals worldwide and is predicted to rise in prevalence over the next two decades [1,2]. The typical concern is the knee's medial compartment osteoarthritis, which causes varus deformity and impairment [3]. Early treatment options are usually nonsurgical, including weight loss, low-impact activity, and physiotherapy. In contrast, surgical options such as high tibial osteotomy, unicompartmental arthroplasty, and total knee arthroplasty are utilized in the later stages [3,4]. For healthy individuals older than 60, arthroplasty is a solid option with good long-term results. However, there are concerns about the lifespan of the implants in younger individuals [3-5].

Medial opening wedge high tibial osteotomy (MOWHTO) is the accepted surgical approach for treating medial compartment osteoarthritis with varus deformity of the knee in younger patients. The open wedge osteotomy surgery is more popular than the closed wedge osteotomy because it does not endanger the peroneal nerve. There is no need to damage the proximal tibiofibular joint or the lateral collateral ligament to achieve accurate correction [5,6]. However, concerns remain regarding selecting appropriate patients, extensive preoperative planning, and precise surgical techniques, all essential for successful outcomes [3]. The success of an osteotomy around the knee depends on the biomechanics, load distribution, and mechanical properties of the osteotomy fixation implants. High tibial osteotomy reallocates weight-bearing stresses, reducing discomfort and delaying disease development [3,7].

Given the significant impact of resource limitations on patient care, it is important to consider how these constraints may affect surgical outcomes. Limited access to advanced medical technologies, rehabilitation services, and postoperative care can result in disparities in treatment effectiveness and recovery among patients in resource-limited settings [8]. Therefore, this study aims to answer the research question: "What are the predictive factors influencing the functional outcomes of MOWHTO in patients with KOA and varus deformity in a resource-limited environment?"

Studies regarding the effectiveness of MOWHTO in improving quality of life and delaying knee replacement among Yemeni patients suffering from isolated medial compartment KOA are lacking. By addressing these factors, this study seeks to contribute to a better understanding of the unique challenges faced by patients and healthcare providers in resource-constrained areas.

## Materials and methods

Study design and patient population

This retrospective, hospital-based observational study was conducted between October 2019 and April 2023 at 48 Modern Hospital and Elite Hospital in Sana'a, Yemen. The primary objective was to evaluate the clinical and radiographic outcomes of MOWHTO in adult patients diagnosed with KOA and varus deformity. Ethical approval was obtained from the Institutional Ethics Committee of 21 September University for Medical and Applied Sciences (approval number: S-98-H-02-F23, approval date: February 9, 2024). The study adhered to the principles of the Helsinki Declaration. Due to its retrospective design, the ethics committee waived the requirement for individual informed consent for chart reviews. All patient data was securely encrypted and maintained confidentially.

Inclusion and exclusion criteria

The study included 102 patients who met specific inclusion criteria, including a diagnosis of KOA with varus deformity amenable to MOWHTO. Exclusion criteria comprised patients who had undergone prior high tibial osteotomy revisions, were more than 60 years of age, or presented with systemic inflammatory arthropathies such as rheumatoid arthritis, gouty arthropathy, or pseudogout, and other systemic comorbidities.

Surgical technique

All surgical procedures were performed under general anesthesia, with prophylactic antibiotics administered preoperatively. A tourniquet was applied to the upper thigh. A standard medial approach was utilized, beginning with a longitudinal incision approximately 1 cm distal to the joint line, midway between the tibial tubercle and the medial border of the tibia. The pes anserinus was retracted to expose the superficial medial collateral ligament, which was partially detached to visualize the medial tibial plateau. Fluoroscopic guidance was employed to insert two Steinmann pins to prevent iatrogenic fractures. The osteotomy was performed distal to the pins, initiated at the medial cortex and directed toward the fibular head using an oscillating saw. The knee was flexed to protect posterior neurovascular structures during the procedure. The osteotomy was completed approximately 1 cm from the lateral cortex using osteotomes. The opening was created, and a hydroxyapatite bone substitute wedge or autogenous iliac crest bone graft was inserted at the posteromedial aspect of the osteotomy site. Correction accuracy was confirmed by a long alignment rod. The osteotomy was stabilized with either a Pudu plate or a proximal tibia buttressing plate. Fixation was achieved with a combination of fully threaded cancellous screws proximally and cortical screws distally. Meticulous hemostasis and deep drainage were employed to minimize postoperative complications. Detailed surgical techniques were described in previous publications [1,2].

Postoperative care

Patients were closely monitored in the recovery room, with attention given to vital signs, neurological status, and neurovascular function. Postoperative care included a period of nothing per os for four to six hours, followed by intravenous fluids, analgesics, and deep vein thrombosis (DVT) prophylaxis. Limb elevation was maintained with a pillow. After a review of neurovascular status and postoperative radiographs, patients were discharged with prescriptions for antibiotics, analgesics, and DVT prophylaxis. Follow-up appointments were scheduled at two weeks postoperatively for suture removal. Gradual weight-bearing with crutches and isometric quadriceps exercises were initiated. Full weight-bearing and progressive quadriceps strengthening exercises were encouraged after six to eight weeks.

Follow-up evaluations

**Figure 1:**
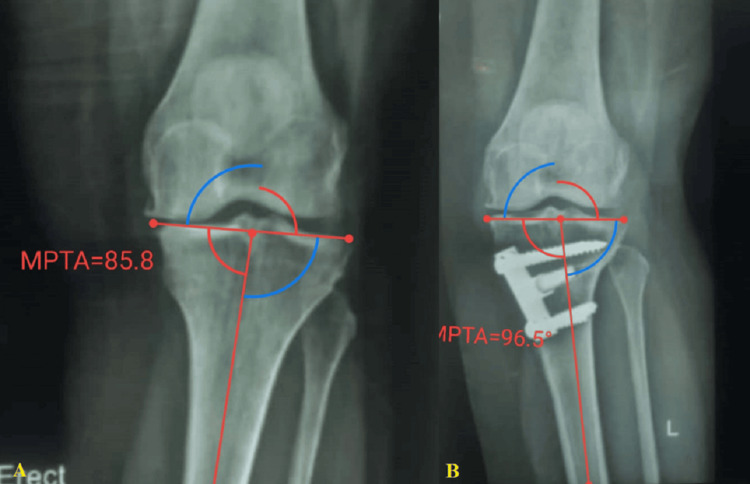
A 53-year-old female patient underwent a left MOWHTO. (A) Preoperative photograph of the entire standing lower limb displaying varus limb alignment (MPTA= 85.8). (B) Postoperative photograph of the fully standing lower limb, demonstrating well-maintained limb alignment (MPTA= 96.5). MOWHTO: medial opening wedge high tibial osteotomy, MPTA: medial proximal tibial angle

Postoperative follow-up assessments were scheduled at three, eight, 12, and 24 weeks, with suture removal occurring at three weeks. Comprehensive clinical and radiological evaluations focused on pain, swelling, tenderness, and range of motion. Radiological follow-up included weight-bearing anteroposterior and lateral views after eight weeks (Figure 1).

Functional outcomes were evaluated utilizing the American Knee Society Functional Score (AKSFS), Clinical American Knee Society Score (CAKSS), and Western Ontario and McMaster Universities (WOMAC) Osteoarthritis Index at the final follow-up [2]. Two experienced surgeons independently performed clinical assessments.

Outcomes

The primary outcomes assessed were the functional and radiological outcomes of MOWHTO. Secondary objectives included the identification of potential factors associated with surgical failure. Surgical failure was defined as conversion to unicompartmental knee arthroplasty (UKA) or TKA.

Data collection and management

A standardized, comprehensive checklist was developed for data extraction from medical records. This checklist encompassed patient demographics, clinical characteristics, and intraoperative, radiographic, and postoperative data. Quality assurance measures were implemented, including thorough training of data abstractors and validation of entries against original medical records. Regular audits were conducted to identify discrepancies, and any inconsistencies were resolved by reevaluating records by a supervising team of healthcare professionals.

Data collected included patient demographics (gender, age, body mass index (BMI), and surgical limb), intraoperative parameters (degree of valgus correction, type of fixation, and graft type), and radiographic parameters (preoperative varus malalignment, Kellgren-Lawrence grading, and evidence of bony union). Two authors independently reviewed radiographic images. Interobserver reliability was assessed using weighted Cohen’s kappa and intraclass correlation coefficient analyses. Postoperative data collected included the incidence of complications and outcomes (success or failure) of MOWHTO.

Sample size and power analysis

The sample size required for this study was calculated using a proportion-based formula, referencing a 13.4% conversion rate from high tibial osteotomies to knee replacement, as reported by Guarino et al. [9]. With a desired margin of error of ±10% and a 95% confidence interval (CI), we determined that a minimum sample size of 45 patients was necessary to obtain reliable estimates for this research.

Statistical analysis

Continuous variables were expressed as means with standard deviations, while categorical variables were expressed as frequencies and percentages. Continuous variables were analyzed using the two-sample t-test, whereas Fisher's exact test was employed for non-normally distributed data. Categorical variables were evaluated using Pearson's chi-square test (χ²), with Fisher's exact test applied when necessary for non-normally distributed data. A multivariate analysis was conducted to identify risk factors associated with surgical failure, adjusting for covariates identified in the univariate analysis. Results were reported as odds ratios (OR) with 95% CIs. Data analysis was performed using SPSS Statistics version 22 (IBM Corp. Released 2013. IBM SPSS Statistics for Windows, Version 22.0. Armonk, NY: IBM Corp.), with a p-value of less than 0.05 considered statistically significant.

## Results

Baseline clinical characteristics

In this study, 102 patients underwent MOWHTO, comprising 45 right knees (44.1%) and 57 left knees (55.9%). The mean duration of postoperative follow-up was 24.4 ± 7.0 months, ranging from 18 to 66 months. The cohort exhibited a mean age of 42.6 ± 8.2 years (range: 25 to 62 years), with a notable female predominance (n=69, 67.6%). The median BMI was 23 kg/m², with an interquartile range of 21 to 34 kg/m². According to the Kellgren-Lawrence classification, disease severity was categorized as follows: grade one was observed in 19 knees (18.6%), grade two in 48 knees (47.1%), grade three in 22 knees (21.6%), and grade four in 13 knees (12.7%). The mean duration of symptoms prior to surgical intervention was 30.9 ± 7.5 months. Within this cohort, 14 patients (13.7%) had a history of prior surgical procedures on the same knee. The buttress plate was employed in 57 cases (55.9%), while the Puddu plate was utilized in 45 cases (44.1%). Notably, MOWHTO was accompanied by autologous anterior iliac crest bone grafting in 38 patients (37.7%) and wedge hydroxyapatite bone substitute in 64 patients (62.7%) (Table 1).

**Table 1:** Demographic and clinical characteristics * Statistically significant result (p<0.05) SD: standard deviation, CI: confidence interval, OR: odds ratio

Variables	Subgroups	Total (N=102)	Success (N=92)	Failure (N=10)	OR (95% CI)	p-value
Age (year)	Mean ± SD	42.6 ± 8.2	41.2 ± 7.3	55.7 ± 4.5	1.41 (1.21-1.75)	<0.001*
Gender	Male	33 (32.4)	30 (32.6)	3 (30.0)	Ref	1
	Female	69 (67.6)	62 (67.4)	7 (70.0)	1.13 (0.29-5.52)	
Laterality	Right side	45 (44.1)	37 (40.2)	8 (80.0)	Ref	0.038*
	Left side	57 (55.9)	55 (59.8)	2 (20.0)	0.168 (0.132-0.219)	
Smoking	No	54 (52.9)	49 (53.3)	5 (50.0)	Ref	1
	Yes	48 (47.1)	43 (46.7)	5 (50.0)	1.141 (0.543-2.433)	
Symptoms duration (months)	Mean ± SD	30.9 ±7.5	30.9 ±7.5	31.2 ±7.8	1.01 (0.91-1.09)	0.902
Body mass index (kg/m²)	Mean ± SD	24.0 ±2.8	23.4 ±2.0	29.6 ±3.7	2.27 (1.59-4.12)	<0.001*
Previous surgery	No	88 (86.3)	80 (87.0)	8 (80.0)	Ref	0.902
	Yes	14 (13.7)	12 (13.0)	2 (20.0)	0.6 (0.243-1.476)	
Severity of cartilage degeneration	Low grade	56 (54.9)	50 (54.3)	6 (60.0)	Ref	0.995
	High grade	46 (45.1)	42 (45.7)	4 (40.0)	0.793 (0.412-1.41)	
Graft type	Autologous anterior iliac crest bone	38 (37.3)	36 (39.1)	2 (20.0)	Ref	0.399
	Wedge hydroxyapatite bone substitute	64 (62.7)	56 (60.9)	8 (80.0)	2.57 (0.60-17.68)	
Implant type	Buttress plate	57 (55.9)	54 (58.7)	3 (30.0)	Ref	0.161
	Puddu plate	45 (44.1)	38 (41.3)	7 (70.0)	3.32 (0.86-16.15)	

Functional outcome

Postoperative functional assessments demonstrated statistically significant enhancements across several metrics. Specifically, the mean AKSFS elevated from 55 ± 5.9 (range: 47 to 67) preoperatively to 87.4 ± 8.0 (range: 54 to 92) at the final follow-up. Similarly, the mean CAKSS improved from 54.7 ± 5.1 (range: 47 to 67) to 87.5 ± 8.0 (range: 54 to 92). In contrast, the WOMAC score notably declined from 64.5 ± 4.0 (range: 51 to 71) to 31.8 ± 6.6 (range: 22 to 55), with all alterations deemed statistically significant (p<0.05 for all comparisons, as detailed in Table 2).

**Table 2:** Comparison of changes in clinical outcomes among preoperative and postoperative functional tests * Statistically significant result (p<0.05). Data were analyzed via paired sample t-test. AKSFS: American Knee Society Functional Score, CAKSS: Clinical American Knee Society Score, CI: confidence interval, WOMAC: Western Ontario and McMaster Universities, MD: mean difference

Variables	Subgroup	Mean± SD (range)	MD	95% CI	p-value
AKSFS score	Preoperative	55.0 ± 5.9 (47.0-67.0)	32.41	35.55-29.28	<0.001*
	Final follow-up	87.4 ± 8.0 (54.0-92.0)			
WOMAC score	Preoperative	64.5 ± 4.0 (51.0-71.0)	32.68	29.41-35.94	<0.001*
	Final follow-up	31.8 ± 6.6 (22.0-55.0)			
CAKSS score	Preoperative	54.7 ± 5.1 (47.0-67.0)	32.82	35.88-29.77	<0.001*
	Final follow-up	87.5 ± 8.0 (54.0-92.0)			

Radiological outcome

Radiological evaluations revealed significant changes, with the hip-knee-ankle angle increasing from 173 ± 4.0° to 186 ± 2.0°, although this change did not reach statistical significance (p=0.1031). The mechanical lateral distal femoral angle recorded a minor increase from 87 ± 3.0° to 87.3 ± 2.0°, although this change did not reach statistical significance (p=0.9615). Conversely, the mechanical medial proximal tibial angle exhibited a significant rise from 84 ± 3.0° to 94.3 ± 4.3°, while the mechanical axis deviation significantly decreased from 31 ± 12.1 mm to 14.2 ± 4.1 mm. Furthermore, the joint line convergence angle saw a reduction from 3.2 ± 2.1° to 2.1 ± 1.1°, with all these variations achieving statistical significance (p=0.0001, p=0.0001, and p=0.0087, respectively). Remarkably, all osteotomy sites demonstrated successful consolidation within 12 months postoperatively, with no reported cases of nonunion (Table 3).

**Table 3:** Comparison of changes in radiological outcomes among preoperative and postoperative functional tests * Statistically significant result (p<0.05) SD: standard deviation, CI: confidence interval, MD: mean difference

Variables	Subgroup	Preoperative	Postoperative	MD (95% CI)	p-value
Hip-knee-ankle angle	Mean ± SD	173 ± 400	186 ±200	-13.00 (-28.70 to 2.70)	0.1031
Mechanical lateral distal femoral angle	Mean ± SD	87 ± 300	87.3 ± 200	-0.3(-12.646 to 12.046)	0.9615
Mechanical medial proximal tibial angle	Mean ± SD	84 ±30	94.3 ± 4.30	-10.3 (12.095 to -8.505)	0.0001*
Mechanical axis deviation (mm)	Mean ± SD	31 ± 12.1	14.2 ± 4.1	16.8 (12.425 to 21.175)	0.0001*
Joint line convergence angle	Mean ± SD	3.2 ± 2.10	2.1 ± 1.10	1.1 (0.288 to 1.912)	0.0087*

Follow-up results and complications

Throughout the follow-up period, four patients (3.9%) encountered a fracture of the lateral cortex, while another five developed pin-tract infections (4.9%). Furthermore, four patients (3.9%) exhibited a loss of deformity correction; notably, one (1.0%) of these individuals experienced premature consolidation of the osteotomy site. Overall, a successful surgical outcome was attained in 92 knees, representing 90.2% of cases, whereas 10 knees (9.8%) were classified as failures. Univariate analyses identified several factors associated with surgical failure, including advanced age (p<0.001), elevated BMI (p<0.001), and left-side laterality (p=0.038). However, when subjected to multivariate logistic regression analysis, significant predictors of failure were narrowed down to advanced age (OR: 1.89; 95% CI: 1.02-3.50, p=0.043) and higher BMI (OR: 2.40; 95% CI: 1.13-5.10, p=0.022) (Table 4).

**Table 4:** Multivariate logistic regression for failure OR: odds ratio, CI: confidence interval * Statistically significant result (p<0.05)

Variables	OR	95% CI	p-value
Age	1.89	1.02-3.50	0.043*
Body mass index	2.4	1.13-5.10	0.022*
Laterality	0.12	0.00-11.40	0.363

## Discussion

This retrospective study aimed to identify predictive factors and assess the functional outcomes of MOWHTO in patients with KOA and varus deformity within a resource-limited setting. The findings confirm MOWHTO as a viable intervention for this patient population, demonstrating positive radiological and functional results. Notably, advanced age and elevated BMI emerged as significant predictors of procedural failure, consistent with the current literature.

The biomechanical disruption inherent in varus knee deformity, characterized by increased stress on the medial compartment, is well-established as a factor in accelerating osteoarthritis progression. MOWHTO addresses this by realigning the lower limb, redistributing weight-bearing forces to the lateral compartment, and reducing pressure on the damaged medial cartilage. This shift in load distribution can potentially decelerate disease progression [10,11].

Surgical planning necessitates careful consideration of the proximal versus distal high tibial osteotomy approaches. While lateral closing-wedge osteotomies may pose a higher risk of neurological complications due to increased soft tissue disruption, open-wedge techniques mitigate these risks [12]. Recent innovations, such as Turi et al.’s open-wedge hemicallotasis using external fixators, exemplify the ongoing efforts to enhance patient outcomes through less invasive strategies [13]. In cases of severe osteoarthritis refractory to conservative management, TKA is often the treatment of choice. However, traditional unilateral arthroplasty may compromise the natural architecture of the knee joint, potentially leading to challenges in prosthetic revision and mobility impairment. Evidence supports the performance of MOWHTO prior to TKA, demonstrating improved knee arthroplasty survival and lower revision rates compared to UKA alone [11,14]. This underscores the clinical significance of MOWHTO in managing advanced KOA and supports the rationale for further research into optimal treatment sequencing to maximize outcomes.

The study cohort presented a mean age of 42 years, with 47.1% of participants aged 31 to 40, a demographic distribution aligning with the literature, which suggests MOWHTO is more prevalent among younger to middle-aged patients [9,15]. A statistically significant association was observed between increasing age and MOWHTO failure, with advanced ages representing barriers to surgical candidacy. This finding is consistent with previous studies identifying advanced age as a risk factor for unfavorable surgical outcomes [9,15-17]. Age-related cellular senescence and accumulated damage can impact knee joint biomechanics, potentially accelerating the progression of osteoarthritis [15]. While Goshima et al. [18] reported no significant effect of age on clinical and radiological outcomes post-surgery, a more nuanced approach to assessing surgical candidacy in older individuals is warranted. Although MOWHTO may yield satisfactory results in patients under 60 years of age, further research is crucial to fully define the safety and efficacy of this procedure in an older population.

Our sample comprised a predominantly female population (67.6%), consistent with previous findings [14,19]. However, we did not find a statistically significant relationship between gender and MOWHTO failure, which is at odds with some reports suggesting female gender as a potential risk factor [20]. This discrepancy highlights the complexity of the interplay of demographic factors in orthopedic surgery and suggests the need for further investigation in larger, more diverse cohorts.

Furthermore, our findings demonstrate a correlation between elevated BMI and an increased risk of MOWHTO failure. The deleterious effects of obesity on surgical outcomes are well-documented [9,15]. Although traditional guidelines have considered a body weight over 90 kg a relative contraindication for high tibial osteotomies [15], recent studies have demonstrated comparable postoperative outcomes in individuals with a BMI exceeding 30 kg/m² [21,22]. Floerkemeier et al. [22] found only slight disparities in PROs between obese and non-obese patients, with no significant differences in complication rates. Similarly, Flecher et al. [21] highlighted that a BMI below 30 kg/m² may confer a protective effect regarding the need for revision procedures. These observations underscore the importance of meticulous patient selection and the potential benefits of preoperative weight reduction, particularly in candidates with obesity.

The role of graft material in postoperative recovery is a subject of ongoing investigation [11,23]. Bone grafting may augment osteotomy stabilization, influence healing dynamics, and potentially alleviate pain, ultimately improving outcomes [23,24]. Some experts advocate for grafting in high-risk patients, particularly those with significant correction requirements or comorbid conditions such as obesity and low bone mineral density [25]. While Zhong et al. [11] suggested that allogeneic bone grafts may expedite healing, long-term differences in union rates have not been statistically significant. The present study did not detect significant differences based on graft type, which may be attributed to the relatively small sample size. This limitation highlights the need for appropriately powered, randomized studies to fully elucidate the influence of various graft materials on surgical outcomes.

Accurate achievement of the desired correction angle during high tibial osteotomies remains a key technical challenge. The optimal angular correction for long-term outcomes is not yet fully established [7]. Insufficient correction can result in less favorable outcomes, which may relate to variability between standing and supine radiographic assessments [26]. Approximately 20% of high tibial osteotomies fail to achieve the intended correction angle [26], potentially reflecting challenges in standardizing radiographic images and the effects of tibial rotation. Furthermore, the relationship between knee alignment and pain levels is complex and poorly understood, suggesting that deformity correction alone may not guarantee symptom relief [5,26,27]. Our study's average preoperative varus deformity was 6.8 ± 0.7°, with a targeted postoperative mechanical axis of 5-6° valgus. Patients achieved a postoperative varus alignment of 5.3 ± 0.4°, demonstrating a 94% success rate. This rate exceeds the 60% success rate reported by Kapila et al. [14] and likely reflects the strict inclusion criteria and the experience of the senior surgeon performing the procedures [28,29]. However, the potential for suboptimal outcomes and the associated impact on patient willingness to pursue further surgical intervention due to financial constraints or personal preferences must be acknowledged.

Recent systematic reviews have assessed the association between postoperative metrics and patient-reported outcomes, focusing on medial proximal tibial slope angle (MPTA) and joint alignment after surgical intervention [30]. One study indicated that a postoperative MPTA exceeding 95° correlated with poorer outcomes on the Knee Injury and Osteoarthritis Outcome Score (KOOS) and the Knee Society Score (KSS) [31]. In agreement with these findings, we documented significant improvements in postoperative functional assessments, with the mean AKSFS improving from 55 ± 5.9 preoperatively to 87.4 ± 8.0 at the final follow-up, and the CAKSS rising from 54.7 ± 5.1 to 87.5 ± 8.0. Moreover, the WOMAC score decreased from 64.5 ± 4.0 to 31.8 ± 6.6; all changes were statistically significant (p<0.05). These results contradict prior reports that did not find significant differences in functional outcomes related to variations in postoperative MPTA or joint alignment angles [32-34]. Our findings suggest that MOWHTO substantially improved overall functional recovery and quality of life despite previous literature indicating limitations in specific patient-reported outcomes. Notably, the significant increases in AKSFS and CAKSS scores after an average follow-up of 22 months echo the findings of Kumar et al. [35], reinforcing the relevance of MOWHTO in contemporary orthopedic practice. Further extended follow-up studies are warranted to assess these functional improvements' durability and cartilage health status. Future research should prioritize longitudinal analyses to comprehensively evaluate MOWHTO’s effectiveness by assessing cartilage status alongside functional outcomes over extended periods.

Clinical implications

This study offers valuable insights into the management of KOA utilizing MOWHTO. Patient selection, prioritizing younger individuals with a normal BMI, is crucial to achieving favorable outcomes. This approach may enhance clinical results and optimize limited healthcare resources. The findings also underscore the need for thorough preoperative discussions that address potential risks and alternative treatments, especially for older patients or those with higher BMIs who may experience surgical challenges. Implementing standardized postoperative care can mitigate complications, and personalized rehabilitation programs can improve patient recovery. Routine follow-up is recommended for patients at higher risk to facilitate early identification of potential complications. Overall, the positive outcomes associated with MOWHTO in a resource-limited setting underscore the need to improve access to this procedure. Continued research efforts are vital to improve success rates and elucidate the factors affecting patient outcomes across diverse populations.

Study limitations

This study is subject to several limitations. The reliance on retrospective data may have compromised data quality due to variability in documentation and record-keeping practices. The retrospective design and small sample size are potential sources of bias, and excluding incomplete records may have introduced selection bias. Furthermore, crucial factors influencing MOWHTO failure, such as educational level and comorbidities, were not recorded. The limited generalizability of the findings, as the study was conducted exclusively in educational hospitals in Yemen, must be acknowledged. Despite these limitations, the study provides valuable insights into prognostic indicators for MOWHTO failure. Future research should prioritize prospective trials with larger sample sizes and extended postoperative follow-up to enhance the robustness and applicability of the findings.

## Conclusions

The results of this study affirm the efficacy of MOWHTO in managing varus medial compartmental KOA, particularly when performed by skilled orthopedic surgeons in early-stage cases involving younger patients with normal BMI. This procedure substantially improves clinical and radiological outcomes, even within resource-constrained settings. The findings underscore the importance of early intervention and individualized treatment planning to optimize patient outcomes and minimize the risk of complications. Further research is needed to investigate the long-term benefits and cost-effectiveness of MOWHTO. Such studies could facilitate the broader adoption of this procedure as an effective treatment option for patients suffering from advanced KOA.
